# Which web to invade? Argyrodine kleptoparasites differentiate amongst architecturally different host webs

**DOI:** 10.3897/BDJ.13.e172146

**Published:** 2025-12-15

**Authors:** Ingi Agnarsson, Fi-Niaina Ramahefarison, Heiða Hlín Matthíasdóttir, Leyla Kudari, Magnús Máni Dagsson, Nína Guðrún Baldursdóttir, Ragnhildur Sara Bergsdóttir, Rakel Bærings Halldórsdóttir, Snorri Björn Magnússon, Snædís Huld Björnsdóttir, Matjaž Gregorič

**Affiliations:** 1 Faculty of Life and Environmental Sciences, School of Engineering and Natural Sciences, University of Iceland, Reykjavik, Iceland Faculty of Life and Environmental Sciences, School of Engineering and Natural Sciences, University of Iceland Reykjavik Iceland; 2 2Department of Entomology, National Museum of Natural History, Smithsonian Institution, Washington DC, United States of America 2Department of Entomology, National Museum of Natural History, Smithsonian Institution Washington DC United States of America; 3 3Department of Entomology, University of Madagascar, Antananarivo, Madagascar 3Department of Entomology, University of Madagascar Antananarivo Madagascar; 4 Research Centre of the Slovenian Academy of Sciences and Arts, Jovan Hadzi Institute of Biology, Ljubljana, Slovenia Research Centre of the Slovenian Academy of Sciences and Arts, Jovan Hadzi Institute of Biology Ljubljana Slovenia; 5 Postgraduate School ZRC SAZU, Ljubljana, Slovenia Postgraduate School ZRC SAZU Ljubljana Slovenia

**Keywords:** Argyrodinae, host choice, kleptoparasitism, kleptotany, spiders

## Abstract

Kleptoparasitism, the theft of resources from another organism, is a widespread foraging strategy amongst animals. Many Argyrodinae cobweb spiders (Theridiidae) are obligatory kleptoparasites that have abandoned web building, relying instead on webs of larger hosts (kleptotany). Theory predicts that kleptoparasites are not randomly distributed amongst host webs and prior studies indicate that web architecture (size and tenure) and prey availability impact kleptoparasite abundance and host choice. This study is the first to examine argyrodine biology in Madagascar and provides an insight into a multi-species endemic community of spider kleptoparasites and their in-situ distribution across contrasting host webs: *Nephilingis* (Nephilidae, large nocturnal orb weavers), *Caripetella* (Pisauridae, large nocturnal sheet web builders) and *Anelosimus* (Theridiidae, small cathemeral social spiders constructing 3D tangle webs). We found a striking diversity of nine kleptoparasite species in six genera that, remarkably, are not conspecific with the five species that were previously known from all of Madagascar. Kleptoparasite species composition and abundance varied across the three hosts: some appeared host-specific, while others are versatile. In general, argyrodine kleptoparasites evidently discriminate amongst hosts, but differ in the degree of host preference. At the community level, we speculate that species assembly into host webs involves a complex interplay between host preference and species competitive hierarchy. Future field research should investigate this system as a community of multiple interacting species to gain a more comprehensive understanding of the rules that may govern the assembly of diverse kleptoparasites into equally variable host webs.

## Introduction

Species interactions range from indirect to direct and from mutually beneficial to hostile. Symbiotic relationship have been defined as “an intimate interaction between different organisms, where at least one of the parties is obligatorily dependent on the association as a part of its life history”. (Leung and Poulin 2008 p. 107). Symbiosis can be mutualistic, commensal or parasitic (Campbell and Reece 2005, Odum and Barrett 2005, Leung and Poulin 2008). Kleptoparasitism is a widespread exploitative foraging strategy involving the theft of extrinsic resources that are of potential value to the host and involves balancing the energy costs of searching for food against the benefits and costs of stealing resources (Brockmann and Dawkins 1979, Vollrath 1979, Elgar 1993, Iyengar 2008, Agnarsson 2025). While facultative kleptoparasitism is common (e.g. Iyengar (2008)), natural selection has engrained this strategy in some groups, where host-selection may be a more critical foraging decision than the exact nature of the stolen resource. Precious little, however, is known about host choice in many kleptoparasitic organisms like spiders (Agnarsson 2025).

Obligate kleptoparasitic spiders are largely kleptotanic, in that they abandon web building and rely on foraging symbiotically in the webs of larger hosts (Vollrath (1984), Vollrath (1987), Whitehouse (1986), Elgar (1993), Grostal and Walter (1997), Whitehouse (2011), Su (2012), Su and Smith (2014), Agnarsson (2025)). The majority of spider kleptoparasites belong to the subfamily Argyrodinae (Theridiidae), a versatile group employing diverse strategies to exploit various host species (Agnarsson 2004). Amongst their hosts are: 1) large nephilid orb weavers such as *Nephila*, *Trichonephila* and *Nephilingis*, that seem to be preferred hosts in most subtropical and tropical environments (Elgar 1993, Whitehouse et al. 2002, Agnarsson 2025); 2) large araneid orb weavers such as *Argiope*, *Cyrtophora* and *Gasteracantha*; 3) large sheet weavers including various agelenids, the pisaurid *Caripetella*, the zoropsid *Tengella* and others; 4) social and colonial spiders (mostly Araneidae, Eresidae and Theridiidae) that build complex silken networks and 5) some smaller species (notably Theridiidae and Linyphiidae) that are locally abundant (Vollrath 1979, Eberhard et al. 1993, Leborgne et al. 1998, Hénaut 2000, Miyashita 2002, Lloyd and Elgar 2006, McCrate and Uetz 2009, Kuntner and Agnarsson 2010, Silva and Sierwald 2013, Agnarsson 2025).

Argyrodinae spiders are speciose (Exline and Levi 1962, Agnarsson 2004, Vanuytven et al. 2024, World Spider Catalog Version 26 2025, Pett et al. 2025) and vary in size, morphology and tactics to obtain resources from host webs (Elgar 1993, Whitehouse 2011) including pilfering small insects, stealing wrapped prey, feeding on digested prey alongside the host, opportunistic predation on the host, its offspring and eggs and even the consumption of host web silk (Whitehouse 1986, Vollrath 1987, Elgar 1993, Miyashita et al. 2004). Their hosts are also diverse, differing in size, innate aggression (solitary vs. communal/social), web architecture and prey choice (Agnarsson 2025). Fundamental foraging and game theories predict that kleptoparasitic species do not distribute randomly amongst potential host webs (MacArthur and Pianka 1966, Gotelli 2008, Iyengar 2008, Gregorič et al. 2024). The limited field observations corroborate these predictions: 1) distribution of kleptoparasites is non-random amongst hosts; 2) large and predictable webs are favoured by kleptoparasites, with large webs, smaller webs and those that have short duration in space and time being disfavoured; 3) larger webs have more kleptoparasite individuals and species and 4) kleptoparasite species range from versatile generalists to relative host specialists (Vinson 1863, Exline 1945, Vollrath 1987, Elgar 1993, Whitehouse et al. 2002, Agnarsson 2003, Agnarsson and Kuntner 2005, Whitehouse 2011, Gregorič et al. 2024). However, most prior studies focus on a single kleptoparasite or a single host species, while many do not identify the kleptoparsites to the species level, complicating comparisons (Whitehouse 1986, Cangialosi 1990, Eberhard et al. 1993, Whitehouse and Jackson 1993, Grostal and Walter 1997, Tso and Severinghaus 1998, Hénaut et al. 2005, Baba et al. 2007, McCrate and Uetz 2009, Moura et al. 2020, Straus and Avilés 2022, Straus and Avilés 2023, Peng et al. 2024, but see Whitehouse (1988)). Amongst the few exceptions, Shinkai (Shinkai 2007) provided a list of host records for eight kleptoparasitic species from Japan, Grostal (Grostal 1999) provided notes on the ecology and the range of hosts of five *Argyrodes* species in Australia, Straus and Avilés (Straus and Avilés 2018) explored the distribution of three morphospecies of *Faiditus* amongst webs of two social and one subsocial *Anelosimus* species and Fernandez-Fournier and Avilés (2018) explored the impact of sociality and web size on the composition of kleptoparasite communities.

**Figure 1.**
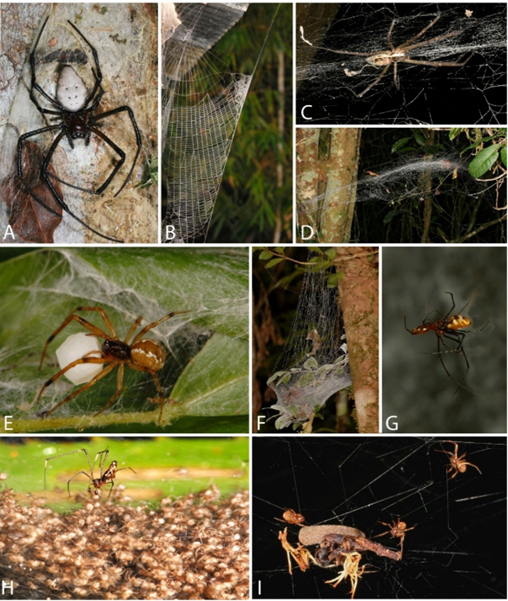
Study system. **A, B** *Nephilingis livida* host, female (A) and vertical orb web (B); **C, D** *Caripetella madagascariensis* host, female in web (C) and horizontal domed sheet web (D); **E, F** *Anelosimus vondrona* Agnarsson & Kuntner, 2005 host, female with egg sac in web (E) and social 3D tangle web (F); **G-I**, kleptoparasites in action. *Famakytta analamazaotra* from this study, pair in copula in *N. livida* web (G), *Famakytta* sp. from Montagne d'Ambre, male approaching freshly hatched *N. livida* spiderlings (H) and *Argyrodes* kleptoparasites stealing prey from *Nephilingis borbonica* (Vinson, 1863) in Réunion (I). Total length of females (front of carapace to spinnerets) *N. livida* ~ 20 mm, *C. madagascariensis* ~ 18-25 mm, *A. vondrona* ~ 5 mm, argyrodines ~ 3-6 mm. Web size (longest axis), *N. livida* orb ~ 40-80 cm high, *C. madagascariensis* sheet ~ 30-60 cm wide, *A. vondrona* basket ~ 10-25 cm width, up to ~ 30 cm tangle above. Photos A, C, D, I by M. Kuntner, B, E-H by I. Agnarsson.

Here, we investigate the distribution of an endemic spider community: multiple argyrodine species amongst three hosts in the small Analamazaotra National Park in Madagascar. The three host species are amongst the most abundant web builders in the Reserve, but contrast starkly in web architecture and behaviour (Fig. 1, see Material and Methods). Our study offers the first insight into argyrodine kleptoparasite biology in Madagascar and is the first to explore in-situ snapshot distribution of a species-rich kleptoparasite community amongst architecturally diverse host webs.

## Material and methods

**Study system**

The hosts all build long lasting webs, but differ in web architecture, size, activity patterns and innate aggression (Fig. 1). *Nephilingis livida* (Vinson, 1863) builds a sticky orb web and *Caripetella madagascariensis* (Lenz, 1886) (Lenz 1886) makes a horizontally domed sheet made of dry silk (Yu et al. 2025), both being nocturnal. *Anelosimus* spiders are subsocial, cathemeral and build three-dimensional tangles of dry silk. Analamazaotra is home to about 10 *Anelosimus* species (Agnarsson et al. 2015) that we sampled indiscriminately, along with a few webs of the architecturally similar subsocial pisaurid *Dendrolycosa* sampled.

**Survey of kleptoparasites in host webs**

**Figure 2.**
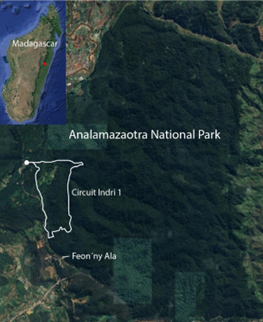
Analamazaotra National Park (25.5 km^2^) is located in eastern Madagascar, see inset map. The edges of the Park are clearly visible by changes in vegetation, evident by dark green forest cover, surrounded by lighter green vegetation and brownish human developments. The research was done along the approximately 2.5 km Indri trail loop (circuit Indri 1) and on the grounds of the Feon'ny Ala cottages. Scale bar in the lower right corner is 1 km.

Analamazaotra National Park is a montane rainforest reserve in eastern Madagascar (25.5 km^2^, between 900-1050 m elevation, centre ca. 18°56'10''S, 48°25'42''E, Fig. 2), where we sampled during 21-28 May 2024. Samples were taken from *Nephilingis livida*, *Caripetella madagascariensis*, *Anelosimus vondrona*, *A. nazariani* Agnarsson & Kuntner, 2005, *A. may* Agnarsson, 2005 and *Dendrolycosa* sp. (Fig. 1, Table 1). The host species are endemic to Madagascar, except *N. livida* that also occurs on northern Indian Ocean islands (Kuntner and Agnarsson 2011). *Caripetella* and *Anelosimus* (Agnarsson and Kuntner 2005, Agnarsson et al. 2015, Agnarsson et al. 2016) were mainly collected along the 2.5 km long Indri 1 trail loop that spans the western portion of the Reserve (Fig. 2). Most *Nephilingis* samples were taken at the forest edge from the grounds of the Feon'ny Ala cottages (Fig. 2).

The kleptoparasities were captured with pooters from ~ 20 webs of *N. livida* and *C. madagascariensis* and placed in live collection vials and used for preliminary translocation experiments described below, before being preserved in ethanol. The entirety of 87 subsocial colonies (75 *Anelosimus* and nine *Dendrolycosa*) were collected in plastic bags and later dissected, with all the web inhabitants preserved in ethanol. Taxonomic identification and molecular studies were subsequently performed at the University of Iceland.

**Translocation experimental pilot**

To examine the feasibility of species translocation for future research we translocated kleptoparasites between *N. livida* and *C. madagascariensis* webs, previously cleared of native kleptoparasites. In 17 translocation experiments, four to seven foreign kleptoparasitic spiders were placed, feet first, on to threads of the outer edge of webs of each cleared host web: 54 native *Caripetella* kleptoparasities on 10 *Nephilingis* webs and 34 native *Nephilingis* kleptoparasities on seven *Caripetella* webs. We then monitored the behaviour and movement of kleptoparasites.

**Morphological identifications and DNA barcodes**

All samples were preserved in 95% ethanol in the field. For details on morphological identification, DNA extraction, sequencing and analyses see Pett et al. (2025).

**Statistical analyses**

To investigate the association between kleptoparasitic species and host web types, we used Fisher’s Exact Test in RStudio (RStudio Team 2020) using simulation with one million replicates (code Suppl. material 1).

## Data resources

Distribution data are provided in Table 1, species descriptions and DNA barcoding data are published in Pett et al. (Pett et al. 2025.

## Results

**Survey of kleptoparasites in host webs and species identification**

A total of 299 argyrodine individuals were sampled (Table 1, Suppl. material 2) belonging to nine species from four known and two new genera, all treated in Pett et al. (2025): Argyrodes cf. argyrodes, *Deelemanella helmscahani*, *Argyrodella ampingamena*, *Famakytta analamazaotra*, *Lokitandroka rabesahala*, *L. ratsimanga*, *L. rinha*, *L. tiana* and *Neospintharus matjazkuntneri*.

**Distribution of kleptoparasitic spiders amongst host webs**

**Figure 3.**
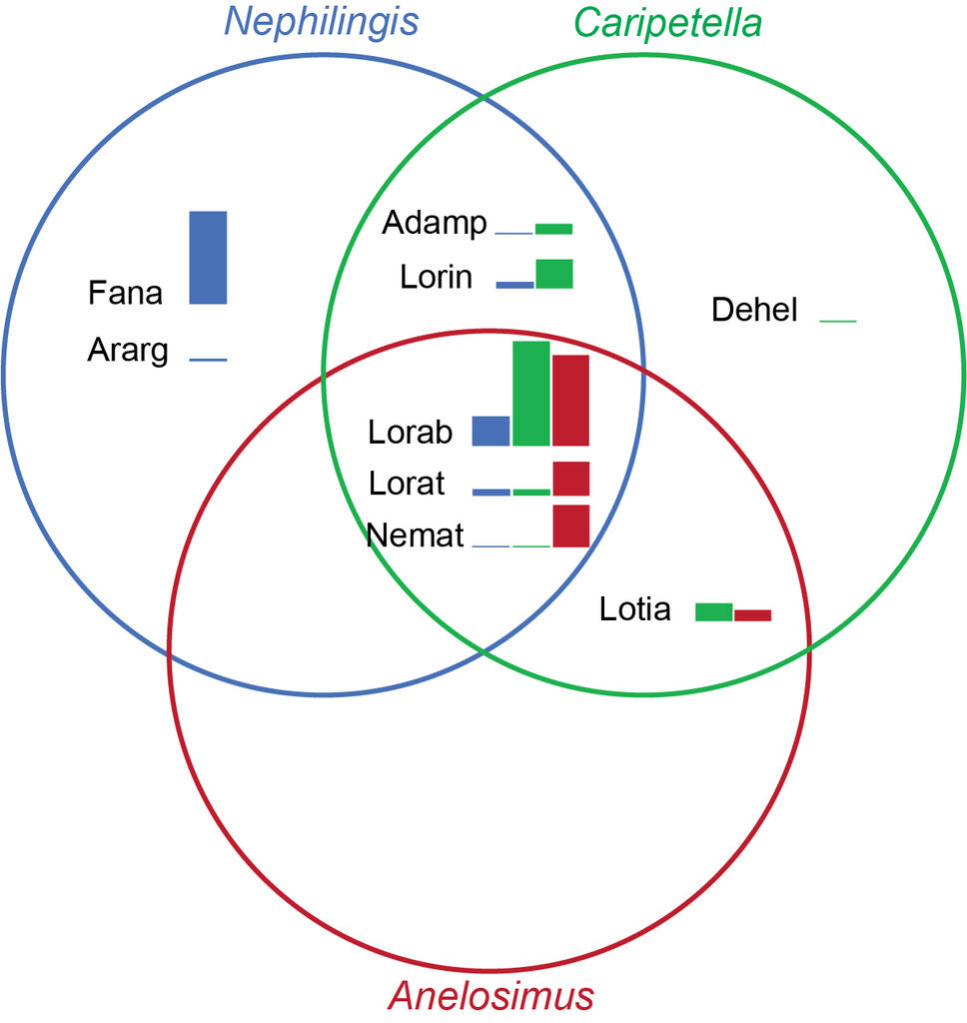
Distribution and overlap of kleptoparasitic species found on different host webs. Fam, Famakytta analamazaotra; Arg, Argyrodes cf. argyrodes; Del, *Deelemanella helmscahani*; Ade, *Argyrodella ampingamena*; Lrab, *Lokitandroka rabesahala*; Lrat, *Lokitandroka ratsimanga*; Lrin, *Lokitandroka rinha*; Ltia, *Lokitandroka tiana*; Neo, *Neospintharus matjazkuntneri*. Bar plots mark the relative abundance of kleptoparasite species for each host web and does not compare their relative abundance amongst host webs.

Species composition and abundance of kleptoparasitic spiders differed amongst host webs (Table 1, Fig. 3, Suppl. material 2). Fisher's Exact Test with simulated p-values based on one million replicates revealed a significant difference in kleptoparasite species association with the three host web types (p < 0.01).

**Translocation experimental pilot**

Of 54 *Caripetella* native kleptoparasitic spiders introduced on to *Nephilingis* webs, 15 (28%) left the web entirely (likely all *Lokiandroka*), 36 (67%) stayed in the host web vicinity, connected to or within 5 cm of the host’s structural thread. Three were lost. *Nephilingis* attacked and killied two introduced kleptoparasites. Of 34 *Nephilingis* native kleptoparasites introduced on to seven *Caripetella* webs, three (all *Famakytta analamazaotra*) (9%) left the web entirely, seven (20.5%) stayed in the vicinity of the host web and 24 (70.5%) remained on the host capture area. *Caripetella* showed no response towards introduced kleptoparasites.

## Discussion

Obligatory kleptoparasitism is a widespread ecological strategy buttressed on the adaptive advantage of stealing resources gathered by another organism, foregoing the cost of independent resource acquisition (Iyengar 2008). In spiders, kleptoparasitic interactions are diverse and involve complex ecological and evolutionary dynamics (Vollrath 1987, Elgar 1993, Whitehouse 2011). Recent research highlights the role of spiders as "superhosts" for obligate kleptoparasites and the versatile strategies spider kleptoparasites utilise to harvest resources (Agnarsson 2025). Host selection by kleptoparasites is driven by traits, such as web size, complexity, persistence and host behaviour, all contributing to the multi-species assembly process (reviewed in Agnarsson (2025)). However, our knowledge of these ecosystems is incredibly sparse: of the about 210 likely kleptoparasitic Argyrodinae species, about half are unknown, most of the remainder are known only by the putative host they were collected from, while 15 or so species are well documented.

Our study exemplifies the value of intense surveys in poorly-known areas. The first inventory of the distribution of argyrodine kleptoparasites amongst host webs in Madagascar revealed a hitherto unknown ecosystem with multiple novel players and interactions. The study is also the first to survey the hosts in question. *Nephilingis livida* is a large, mostly nocturnal spider constructing a huge and sticky vertical orb web (Gregorič et al. 2011). Despite the size and ubiquity of *N. livida*, the only kleptoparasite that has been recorded in its web is *A. argyrodes* (see Agnarsson (2025)). *Caripetella madagascariensis* is an exclusively nocturnal giant nursery web spider that was documented only recently in scientific literature to construct a prey capture web (Yu et al. 2025). Her web, one of the world’s largest horizontal domed sheets, is thus an unstudied ecosystem. The cathemeral *Anelosimus* and *Dendrolycosa* form subsocial spider communities that construct three-dimensional tangle webs, whose numerous inhabitants — a mother and her offspring — are barely larger than the kleptoparasites (Agnarsson and Kuntner 2005). While *Argyrodes* sp. and *Neospintharus* sp. have been haphazardly recorded from Malagasy *Anelosimus*, surveys are lacking and no kleptoparasites have been documented from *Dendrolycosa*.

We found a high diversity of nine argyrodine kleptoparasite species, all of which are new to science (see Pett et al. 2025). This discovery aligns with prior knowledge of Analamazaotra as rich in spider diversity, for example, as a world hotspot of the cosmopolitan *Anelosimus* spiders (Agnarsson et al. 2015, Agnarsson et al. 2016) and old world *Caerostris* spiders (Gregorič et al. 2011). The study area represents less than 1/200,000^th^ of this vast island, yet we sampled double the diversity of kleptoparasitic argyrodine spiders hitherto known from all of Madagascar, underscoring the remarkable biodiversity of Madagascar and how even short-term studies can reveal new insights into this globally distributed ecosystem.

Kleptoparasite species composition and abundance differed amongst host webs. Some kleptoparasites appear to be highly selective, for example, three out of ten species occurred exclusively in webs of a single host, including the most common kleptoparasite in *Nephilingis* webs, *Famakytta analamazaotra* (Fig. 3). Even amongst species that utilised all three hosts (e.g. *Lokitandroka rabesahala*, *L. ratsimanga*), their relative abundances generally tended towards a particular host species or web type (Fig. 3). From the perspective of host species, *Caripetella* and *Nephilingis* shared an argyrodine community of five species, but in different abundances and each hosted additional species not found in the other. These large webs, thus, represent distinct habitats for kleptoparasites. Similarly, while each of these share a community of four argyrodine species with the sub-social *Anelosimus*/*Dendrolycosa* webs, the latter stand out in harboring relatively few species, yet hosting the vast majority of *Neospintharus matjazkuntneri*.

Detailed studies have been undertaken on a dozen or so kleptoparasite species, for example, *Argyrodes antipodianus* (Whitehouse 1986, Whitehouse 1988, Whitehouse 2011), *Faiditus ululans* (e.g. Cangialosi (1990)) and *Neospintharus trigonum* (e.g. Larcher and Wise 1985, Cangialosi 1997); or sometimes on co-existing species pairs like *Argyrodes elevatus* and *Faiditus caudatus* (Vollrath 1979, Vollrath 1984, Vollrath 1987). The kleptoparsitic communities of certain regions (e.g. Shinkai (2007)) like Japan and a few host species are relatively well studied, including *Cyrtophora citricola*, *Trichonephila clavipes* and *Metepeira incrassata* (Agnarsson 2025). However, understanding host and kleptoparasite interactions and species assembly at the community level is at its infancy. Our study is amongst very few that provide a snapshot of the distribution of a diverse and endemic community of kleptoparasites across contrasting webs of similarly endemic hosts within a geographically restricted area. Our findings align with the most inclusive prior study, where Fernandez-Fournier and Avilés (Fernandez-Fournier and Avilés 2018) found that, amongst two social and two solitary spider species, host species traits, like social structure and web architecture, significantly influence their kleptoparasite community. Our findings contribute to an emerging picture of dynamic kleptoparasite communities influenced by the traits of their potential hosts.

Our data do not indicate whether all kleptoparasite species found in a particular host web use these as preferred resource sites (Whitehouse 2011). Regardless, it seems clear that different host species provide distinct habitats. For example, only 4/9 kleptoparsite species were documented in *Anelosimus* webs, all in low abundance, averaging less than one individual per web. The relatively small size of *Anelosimus* webs may restrict kleptoparasite abundance and, no doubt, kleptoparsites operating in social webs inhabited by numerous, ever active, small juvenile spiders, may require distinct behavioural adaptations. Social groups of co-feeding hosts may also offer few opportunities of stealing prey, but possibilities to predate on juvenile hosts. Indeed, *Neospintharus matjazkuntneri* that was predominantly found in *Anelosimus* webs pertains to a genus well known for araneophagy (Larcher and Wise 1985, Cangialosi 1997). In our translocation pilot, we also observed three *F. analamazaotra*, not found in *Caripetella* webs in nature, being the only translocated kleptoparsites to immediately leave a *Caripetella* web when introduced, suggesting this species may rapidly detect non-preferred host webs.

The observed kleptoparasite distribution results from microhabitat (web) choice, ranging from minimal preference (e.g. the versatile *L. rabesahala*), to astutely choosy species like *F. analamazaotra* - the largest kleptoparasite in this study that may prefer nephilid hosts likely to catch relatively large prey (Nyffeler and Gibbons 2022). Alternatively, the distribution of kleptoparasites may result from competition. *Famakytta analamazaotra* may, for example, have competitive advantage due to size and occupy the best habitat patches (Nephilidae is a globally preferred argyrodine host), while the small and delicate *Neospintharus matjazkuntneri* may prefer social nests or may be competitively inferior to other argyrodines and, thus, restricted to smaller and less favourable habitats.

As more community level studies accumulate, our study continues to cast doubt on prior attempts to fit argyrodines into binary bins, for example, host generalist vs. specialist. While a tempting simplification, such boxes have proven to be ill-fitting as data accumulate, even for relatively choosy kleptoparasites (Vollrath 1984, Larcher and Wise 1985, Whitehouse 1986, Downes 1995, Whitehouse et al. 2002, Baba et al. 2007, Whitehouse 2011, Su 2012, Su and Smith 2014, Agnarsson 2025, Cowles and Agnarsson 2025). Instead, argyrodines may better be characterised as generally capable of discriminating amongst host webs and these webs might offer them resources of diverse values and/or types. Prior research has established that web architecture and prey availability are important factors in individual´s host choice (Larcher and Wise 1985, Whitehouse 1986, Cangialosi 1990, Eberhard et al. 1993, Miyashita 2002, Koh and Li 2006, Baba et al. 2007). However, in diverse communities of kleptoparasite and host species, underlying mechanisms of host choice are, no doubt, more complex and interactive (Agnarsson 2025). Therefore, to understand the distribution of kleptoparasitic species in ecosystems, we need to address host choice at the level of community assembly.

## Conclusions

We find a diverse and previously undocumented kleptoparasite community in a rapid survey in Madagascar, showing that multiple argyrodine species distribute non-randomly amongst three types of host webs. Host choice or, more generally, the community assembly of kleptoparasite species into host webs, likely results from an interplay of host-specialisation and interspecific competition. We predict that host-specific chemicals (likely pheromones in silk) stemming from an existing system guiding male spiders to conspecific female webs, have played a role in the evolution of web kleptoparasites. Fundamental field research is needed on kleptoparasites community assembly and laboratory experiments on chemical cues that play a role in kleptoparasite web detection and choice.

## Supplementary Material

72FD9F9D-8B77-5D97-952A-36982444FC21 10.3897/BDJ.13.e172146.suppl1 Supplementary material 1 Which Web to Invade: Supplementary Material for Statistical Analyses Data type script for statistical analyses Brief description This script details the steps used to perform statistical analyses of the contingency table examining the association between kleptoparasitic species and host web types. The tests include Chi-Square and Fisher's Exact Test for data with low expected values. File: oo_1407716.pdf https://binary.pensoft.net/file/1407716 Ingi Agnarsson, Heiða Hlín Matthíasdóttir, Nína Guðrún Baldursdóttir, Leyla Kudari, Magnús Máni Dagsson, Ragnhildur Sara Bergsdóttir, Rakel Bærings Halldórsdóttir, Fi-Niaina Ramahefarison, Snorri Björn Magnússon, Snædís Huld Björnsdóttir, Matjaž Gregorič

96F849B2-1BB1-5BA2-8152-855C1E05E4D5 10.3897/BDJ.13.e172146.suppl2 Supplementary material 2 Table S1 Data type Table Brief description Image of Table 1 with colours highlighting species abundances amongst host webs. File: oo_1411597.jpg https://binary.pensoft.net/file/1411597 Agnarsson et al.

## Figures and Tables

**Table 1.** The distribution and abundance of the ten kleptoparasite species found amongst the studied three host webs (Faana, *Famakytta analamazaotra*; Ar, Argyrodes cf. argyrodes; Del, *Deelemanella helmscahani*; Ade, *Argyrodella ampingamena*; Lrab, *Lokitandroka rabesahala*; Lrat, *Lokitandroka ratsimanga*; Lrin, *Lokitandroka rinha*; Ltia, *Lokitandroka tiana*; Neo, *Neospintharus matjazkuntneri*). See Suppl. material 2 for cell-shading that marks the relative abundance of kleptoparasite species for each host web and does not compare their relative abundance amongst host webs.

**Host**	**Fam**	**Arg**	**Del**	**Ade**	**Lrab**	**Lrat**	**Lrin**	**Ltia**	**Neo**	**Total**
* Nephilingis *	72 66.1%	2 1.8%	0 0%	1 0.9%	23 21.1%	5 4.6%	5 4.6%	0 0%	1 0.9%	109
* Caripetella *	0 0%	0 0%	1 0.8%	8 6.3%	78 61.4%	5 3.9%	21 16.5%	13 10.2%	1 0.8%	127
* Anelosimus *	0 0%	0 0%	0 0%	0 0%	32 50.8%	12 19%	0 0%	4 6.3%	15 23.8%	63
