# Supplementary material for: Which web to invade? Argyrodine kleptoparasites differentiate amongst architecturally different host webs
Source: Biodivers Data J. 2025 Dec 15;13:e172146. doi: 10.3897/BDJ.13.e172146 (PMC12723393; doi:10.3897/BDJ.13.e172146)
Supplement: Supplementary material 1 — Which Web to Invade: Supplementary Material for Statistical Analyses [file bdj-13-e172146-s001.pdf]

Supplementary Material for Statistical Analyses
Software Used: RStudio
Version: 2023.09.1+494

This script details the steps used to perform statistical analyses of the contingency table examining the association between kleptoparasitic species and host web types. The tests include Chi-Square and Fisher's Exact Test for data with low expected values.

```
# Load necessary libraries for data handling and analysis
library(readxl)        # For reading Excel files
library(exact2x2)      # For Fisher's Exact Test
library(MASS)          # For handling statistical operations, if needed

# Load the contingency table from the relevant Excel file
# Replace "path/to/your/data.xlsx" with the actual path to your file
contingency_table <- read_excel("path/to/your/data.xlsx")
# Or use CSV for easier import
# Replace "path/to/your/data.csv" with the actual path to your file
contingency_table <- read.csv("path/to/your/data.csv")

# Print the contingency table to confirm it's loaded correctly
print(contingency_table)

# Check expected values in the contingency table
expected_values <- chisq.test(contingency_table)$expected
print(expected_values)

# Identify cells with expected values less than 5 which may invalidate Chi-Square
low_expected <- ifelse(expected_values < 5, TRUE, FALSE)
print(low_expected)

# Perform the Chi-Square test if all expected values are adequate
if(all(!low_expected)) {
  chi_test_result <- chisq.test(contingency_table)
  print(chi_test_result)
}

# If expected values are too low, proceed with Fisher's Exact Test with simulation
if(any(low_expected)) {
  fisher_test_result <- fisher.test(x = contingency_table, simulate.p.value = TRUE, B = 1e6)
  print(fisher_test_result)
}

# Note: The code is specifically for performing Fisher's Exact Test with a contingency table.
# Adjust file paths, parameters, and error handling as needed based on your dataset and analysis specifics.
```
