# Supplementary figures and images for: Which web to invade? Argyrodine kleptoparasites differentiate amongst architecturally different host webs
Source: Biodivers Data J. 2025 Dec 15;13:e172146. doi: 10.3897/BDJ.13.e172146 (PMC12723393; doi:10.3897/BDJ.13.e172146)

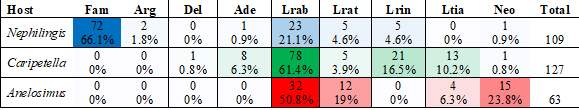

Supplement: Supplementary material 2 — Table S1 [file bdj-13-e172146-s002.jpg]
